# Vascular Parkinsonism and cognitive impairment: literature review, Brazilian studies and case vignettes

**DOI:** 10.1590/S1980-57642012DN06030005

**Published:** 2012

**Authors:** Thiago Cardoso Vale, Maira Tonidandel Barbosa, Paulo Caramelli, Francisco Cardoso

**Affiliations:** 1 Neurology Division, University Hospital, Faculty of Medicine, Federal University of Minas Gerais (UFMG), Belo Horizonte MG, Brazil.; 2 Internal and Geriatric Medicine, Internal Medicine Department, Faculty of Medicine, Federal University of Minas Gerais (UFMG), Belo Horizonte MG, Brazil.

**Keywords:** vascular parkinsonism, vascular cognitive impairment, vascular dementia, Parkinson's disease, diffuse white-matter lesions

## Abstract

Vascular Parkinsonism (VP) is a form of secondary Parkinsonism resulting from cerebrovascular disease. Estimates of the frequency of VP vary greatly worldwide; 3% to 6% of all cases of Parkinsonism are found to have a vascular etiology. In a Brazilian community-based study on Parkinsonism, 15.1% of all cases were classified as VP, the third most common form, with a prevalence of 1.1% in an elderly cohort. Another Brazilian survey found a prevalence of 2.3% of VP in the elderly. VP is usually the result of conventional vascular risk factors, particularly hypertension, leading to strategic infarcts of subcortical gray matter nuclei, diffuse white matter ischaemic lesions and less commonly, large vessel infarcts. Patients with VP tend to be older and present with gait difficulties, symmetrical predominant lower-body involvement, poor levodopa responsiveness, postural instability, falls, cognitive impairment and dementia, corticospinal findings, urinary incontinence and pseudobulbar palsy. This article intends to provide physicians with an insight on the practical issues of VP, a disease potentially confounded with vascular dementia, idiopathic Parkinson's disease, dementia with Lewy bodies and other secondary causes of Parkinsonism.

## Abstract (pt)

Parkinsonismo vascular (VP) é a forma secundária da síndrome parkinsoniana resultante de doença cerebrovascular. Há grande variação das estimativas de frequência em estudos mundiais, sendo que em média 3% a 6% de todos os casos de parkinsonismo têm a etiologia vascular. Em um estudo brasileiro de base comunitária sobre parkinsonismo, 15,1% de todos os casos foram classificados como VP, que foi a terceira causa mais comum, com uma prevalência de 1,1% em uma coorte de idosos. Outro estudo brasileiro encontrou uma prevalência de 2,3% de VP também em idosos. VP usualmente resulta de fatores de risco vasculares como a hipertensão, levando a infartos estratégicos nos núcleos da base, lesões isquêmicas difusas da substância branca subcortical e menos comumente, infartos de grandes vasos. Os pacientes com VP geralmente são mais idosos e apresentam dificuldades para a marcha, envolvimento simétrico predominante em membros inferiores, resposta pobre à terapêutica com levodopa, instabilidade postural e quedas, comprometimento cognitivo e demência, sinais de acometimento corticoespinhal, incontinência urinária e paralisia pseudobulbar. Este artigo apresenta algumas informações práticas sobre o VP, uma condição neurológica potencialmente confundida com demência vascular, doença de Parkinson idiopática, demência com corpos de Lewy e com outras causas de parkinsonismo.

## INTRODUCTION

Vascular Parkinsonism (VP) is a form of secondary Parkinsonism resulting from cerebrovascular disease. Diagnosing VP is often problematic in daily clinical practice not only for general neurologists but also for movement disorders specialists, and has remained a controversial clinical concept. The clinical diagnosis of VP is supported by radiological studies which have evolved over the last two decades from computed tomography (CT) to magnetic resonance imaging (MRI) and dopamine transporter single photon-emission CT scan (DAT-SPECT).^1^ Although the clinical and radiological features of VP are widely known, no consensus exists with regard to its pathophysiology, diagnostic criteria and treatment. This review intends to provide physicians with an insight on the practical issues of VP, a disease potentially confounded with vascular dementia (VD), idiopathic Parkinson's disease (PD), Lewy body dementia (LBD) and other secondary causes of Parkinsonism.

## HISTORY

VP was first described by Critchley in 1929.^2^ The clinical presentation of what Critchley coined "arteriosclerotic Parkinsonism" included rigidity, fixed faces, and short stepping gait as the main clinical signs. Pseudobulbar features, dementia, urinary incontinence, pyramidal or cerebellar signs were considered additional features. Critchley failed to provide any pathological correlation and subsequently the validity of his concept was called into question until CT, and later MRI, became available.^3^ Discrete basal ganglionic and diffuse subcortical white matter lesions were identified in patients with Parkinsonism that were clinically different from PD. In 1981, Critchley renamed the condition as "arteriosclerotic pseudo-Parkinsonism",^4^ after several clinical studies in the 1960s and 1970s had shown no relation between arteriosclerosis and PD.^5-7^ In 1987, Thompson and Marsden described 12 cases of Binswanger's disease with symmetrical hypodensities in cerebral white matter on CT, Parkinsonian signs and gait features that closely resembled those in the cases described by Critchley.^8^ They termed these cases as having "lower-half Parkinsonism". Around the same time, it was debated that Binswanger's disease was not in fact a "disease" and that this eponym denoting cerebral symmetrical white matter hypodensities on CT should be replaced by "leukoaraiosis".^9,10^ In 1989, Fitz-Gerald and Jankovic coined the term "lower body Parkinsonism"^11^ and later, in 1999, Winikates and Jankovic^12^ proposed the first clinical criteria for the diagnosis of VP. However, more strict clinical criteria were proposed only in 2004, after the first clinico-pathological study on VP.^13^

## EPIDEMIOLOGY

VP has been reported to account for 2.5% to 5.0% of all cases of Parkinsonism in various population-based studies and clinical series.^1^ Estimates of the frequency of VP vary greatly worldwide. These discrepancies are largely explained by variations in case definition and methodology. Considering only those studies with either imaging or pathological support for the diagnosis, 3% to 6% of all cases of patients with Parkinsonism are found to have a vascular etiology.

In the Rotterdam study, a prospective population-based cohort study of 132 people aged 55 years or more with Parkinsonism, 5% fulfilled criteria for VP.^14^ In a population-based study in Spain, involving 5,278 elderly subjects, VP was diagnosed in only three subjects (2.5% of Parkinsonism cases).^15^ In a joint analysis of five community surveys of Europe, the EUROPARKINSON (European Community Concerted Action on the Epidemiology of Parkinson's disease) collaborative study, VP accounted for 3% of the total cases of Parkinsonism.^16^

In a Swiss hospital-based autopsy study of 261 individuals with Parkinsonism, PD was diagnosed in 62.2%, followed by VP in 8.8%, Alzheimer-type pathology of the substantia nigra in 8%, progressive supranuclear palsy in 4.2%, multiple system atrophy in 2.3%, corticobasal degeneration in 1.2% and postencephalitic Parkinsonism in 2.7%. These authors noted that PD was greatly clinically overdiagnosed, with VP and Alzheimer-type pathology being the most frequent confounding conditions.^13^

## BRAZILIAN EPIDEMIOLOGICAL STUDIES

In Brazil, Cardoso et al.^17^ reported the presence of VP in 4.7% of 338 patients followed up at a tertiary-care specialized movement disorder unit. More recently, Munhoz et al.^18^ diagnosed VP in 3.9% of patients in a large clinical-based series of 1,528 patients with Parkinsonism.

In a Brazilian community-based survey (Bambuí Study), Barbosa et al.^19^ found 86 cases of Parkinsonism among 1,186 participants (aged 64 and older). The prevalence rate in this group was 7.2% for all types of Parkinsonism. The most frequent causes were PD and drug-induced Parkinsonism, with prevalence rates of 3.3% (n=39) and 2.7% (n=32), respectively. VP was the third most frequent etiology, diagnosed in 13 cases, with a prevalence rate of 1.1% (95% CI, 0.4 to 1.8). A higher prevalence was seen for men with advanced age (p=0.01) but not for women (p=0.33) while there was no gender difference in prevalence (Table 1).^19^ The high number of VP patients (15.1% of all cases of Parkinsonism) could be related to the well-established vascular risk factors such as increased age, hypertension, diabetes, smoking, as well as the high prevalence of Chagas disease in the Brazilian elderly population. Chagas disease, in its chronic form, can be associated with ischemic stroke, cardiac arrhythmia and heart failure.

**Table 1:** Prevalence of VP in the elderly cohort of Bambuí, Brazil.^19^

Age (years)	Women			Men			Total	
	**N. of cases**	**Prevalence %**		**N. of cases**	**Prevalence %**		**N. of cases**	**Prevalence %**
64-69	2	0.7%		0	-		2	0.4%
70-74	1	0.5%		0	-		1	0.3%
75-79	3	2.3%		3	3.7%		6	2.8%
80-84	0	-		2	4.8%		2	1.7%
>84	1	2.3%		1	3.7%		2	2.9%
Total	7	1.0%		6	1.3%		13*	1.1%

* N=13 in a population of 1,186 ≥ 64 year-old participants.

Roriz-Cruz et al.^20^ conducted a survey on Parkinsonian syndromes among 454 elderly people living in two communities (Estancia Velha and Charqueadas) from Rio Grande do Sul, Brazil's Southernmost state. A total of 42 (9.9%) subjects were found to have Parkinsonian syndrome. Thirteen cases (3.0%) were diagnosed as PD, 16 cases (3.7%) as drug-induced Parkinsonism and 10 cases (2.3%) as VP, corresponding to 23.8% of all cases of the syndrome. Of note, prevalence of hypertension (84.6%) and clinically manifested stroke (10.1%) were higher in this elderly community as compared with reports from most regions of the world for this age-group.^20^

## PATHOPHYSIOLOGY

The exact pathophysiological mechanisms leading to VP are currently unknown. VP is the result of conventional vascular risk factors, particularly hypertension, leading to strategic infarcts of subcortical gray matter nuclei, diffuse white matter ischemic lesions (DWML) and, least commonly, large vessel infarcts.^1,21-23^

Clinico-radiological^11,22,24^ and clinico-pathological studies^25,26^ have shown statistically more cerebral infarcts in VP than in neurologically normal subjects with PD. DWML may cause Parkinsonism due to damage to the net of thalamocortical loops, which decreases the ultimate influence of basal ganglia on higher centers of motor planning and execution. Strategic infarcts, such as those located at the substantia nigra, striatum or ventroanterior and ventrolateral nuclei of the thalamus, would be expected to cause Parkinsonism by disrupting the putamino-pallido-thalamic loop.^1,8^

## CLINICAL FEATURES AND DIAGNOSTIC CRITERIA

The clinical picture of VP is heterogeneous. Several clinical features have been described relating brain vascular lesions to Parkinsonism. A clinical diagnosis of VP remains difficult to establish as infarction of the basal ganglia and deep white matter occur frequently in elderly people who do not have Parkinsonism^27^ and vascular lesions are a common incidental finding in pathologically-confirmed PD.^28^ Hence, a large proportion of patients with late-onset PD have some degree of white-matter change on CT/MRI brain scans.

Given its clinical heterogeneity, Fénelon and Houéto^29^ stratified VP into four types according to clinical manifestations:

[A] VP manifesting in a manner identical to PD;
[B] unilateral Parkinsonism following contralateral vascular lesion;
[C] "atypical" Parkinsonian syndromes;
[D] "Parkinsonian" gait disorders.

DWML, also known as leukoaraiosis, are commonly observed on imaging studies in older adults, and may present as signal hyperintensities on T2-weighted MRI studies.^30^ Age-associated DWMLs are associated with balance impairment, mobility and cognitive deficits in otherwise healthy elderly individuals.^31,32^ *In vivo* imaging studies show DWMLs to be present in 30-55% of patients with PD.^30,33,34^ Some studies also suggest that DWMLs are more common in patients with PD than in normal elderly individuals.^24,35^ The association between comorbid DWMLs and PD most consistently manifests in impairment of axial motor symptoms and executive functions, determining a subtype of a more rapidly evolving and aggressive PD. This subtype can be easily confused with VP and indeed such patients might have contributed to raising the epidemiologic statistics for VP.

There are many overlapping features that pose a significant challenge in diagnosing VP. Many cases of pathologically-confirmed VP reported in the literature were diagnosed as PD due to several highly suggestive clinical features, including levodopa responsiveness. Zijlmans et al.^36^ reported a good or excellent response to levodopa in 12 out of 17 patients with pathologically-confirmed VP. All the same, patients with typical features of PD who had evidence of cerebrovascular disease on neuroimaging were diagnosed as PD with coexistent cerebrovascular disease, but post-mortem studies revealed no Lewy-bodies and therefore VP was the most likely diagnosis. This uncertainty and difficulty in diagnosing Parkinsonian syndrome is exemplified by the study of Horvath et al.^37^ who investigated the clinical accuracy of diagnosis with the pathological gold standard method. The overall diagnostic accuracy in their sample was 63.4%. For PD, it was 71.2% for the entire study period and increased during the ensuing decades, reaching 85.7% in the last decade. Clinical misdiagnoses included nine cases with unspecified Parkinsonian syndrome, one case of postencephalitic Parkinsonism, one case of progressive supranuclear palsy, three cases of VP and one case of drug-induced Parkinsonism.

Unilateral Parkinsonian syndromes caused by contralateral vascular lesions represent "pure" VP, whose criteria include the appearance of unilateral Parkinsonism following an ischemic and haemorrhagic stroke in the substantia nigra, thalamus or at strategic locations that disrupt the striatopallidal loop.^38^ Striatal or striatopallidal ischemic lesions have been more frequently observed than substantia nigra lesions.^29,38,39^

The first clinical criteria were proposed by Winikates and Jankovic in 1999.12 Parkinsonism was defined as the presence of at least two of the four cardinal signs: tremor at rest, bradykinesia, rigidity and loss of postural reflexes. Evidence of vascular disease was assessed using a vascular rating scale. Two points were given for pathologically or angiographically-proven diffuse vascular disease; one point for onset of Parkinsonism within one month of a clinical stroke; one point for history of two or more risk factors for stroke; and one point for neuroimaging evidence of vascular disease in two or more vascular territories. Patients with Parkinsonism and a vascular score of two or more were categorized as having VP.

These criteria were based on a retrospective, cross-sectional and hospital-based clinical study which compared 69 VP with 277 PD subjects. No clinico-pathological correlation was evident. Patients with VP were significantly older and presented with prominent gait difficulties, symmetrical predominant lower-body involvement, less levodopa responsiveness, postural instability, falls, dementia, corticospinal findings, urinary incontinence and pseudobulbar palsy.^12^ Risk factors for stroke were significantly more common in the VP group, with the greatest differences being in hypertension and heart disease, followed by diabetes and hyperlipidemia and smoking.

In the Bambuí Study,^19^ VP was defined according to the Winikates and Jankovic criteria,^12^ and was based on the presence of at least two of the following findings: history of repeated strokes with abrupt onset and stepwise progression of Parkinsonism features, hypertension, broad-based rigid gait, and widespread pyramidal signs. Diagnosis was reinforced by CT or MRI with vascular lesions in white matter, basal ganglia or the cerebral hemispheres.

In 2004, Ziljmans et al.^13^ proposed new clinical criteria for diagnosing VP based on their clinico-pathological findings (Table 2).

**Table 2:** Possible criteria for the clinical diagnosis of VP.^13^

**Parkinsonism**	**Cerebrovascular disease**
Bradykinesia (slowness of initiation of voluntary movement with progressive reduction in speed and amplitude of repetitive actions in either upper or lower limbs, including the presence of reduced step length) and at least one of the following: rest tremor, muscular rigidity, or postural instability not caused by primary visual, vestibular, cerebellar or proprioceptive dysfunction.	Defined by evidence of relevant cerebrovascular disease on brain imaging (CT or MRI) or the presence of focal signs or symptoms that are consistent with stroke.
**A relationship between the above two disorders**	**Exclusion criteria for VP**
In practice: [1] acute or delayed progressive onset with infarcts in or near areas that can increase basal ganglia motor output (GPe or substantia nigra pars compacta) or decrease thalamocortical drive directly (VL of the thalamus, large frontal lobe infarct). Parkinsonism at onset consists of a contralateral bradykinetic rigid syndrome or shuffling gait, within 1 year after a stroke (VPa). [2] insidious onset of Parkinsonism with extensive subcortical white matter lesions, bilateral symptoms at onset, and the presence of early shuffling gait or early cognitive dysfunction (VPi)	History of repeated head injury, definite encephalitis, neuroleptic treatment at onset of symptoms, presence of cerebral tumor or communicating hydrocephalus on CT or MRI scan, or other alternative explanation for Parkinsonism.

GPe: globus pallidus externa; VL: ventro-lateral nuclei; VPa: vascular Parkinsonism with an acute onset; VPi: vascular Parkinsonism with a insidious onset.

A recent systematic review of features that helps differentiate VP from PD comprised 25 articles, seven of which described clinical features only. In the review, diagnostic criteria for VP varied across studies and other causes of Parkinsonism were excluded. Mean age at symptom onset in patients with VP was four to ten times higher than in patients with PD. The mean duration of symptoms at the time of presentation was shorter for VP compared to PD. Patients with VP more commonly presented with symmetrical gait difficulties, postural instability, falls, dementia, pyramidal signs, pseudobulbar palsy and urinary incontinence. Patients with PD were more rigid and tremulous, and tended to have more hypokinesia or bradykinesia. Vascular risk factors were more common in VP than in PD.

The VADO, an Italian multicentre study,^40^ not included in the above systematic review, showed that VP and PD groups did not differ for gender, age, disease duration, total daily medication dosage, or vascular risk factors. VP was diagnosed based on Zijlmans et al.^13^ criteria. The only vascular risk factor recorded more frequently in VP than PD was diabetes with only a trend toward hypertension. Five studies reported a significantly poorer L-dopa response in VP than in PD.

## COGNITIVE IMPAIRMENT AND DEMENTIA IN VASCULAR PARKINSONISM

Unlike in PD, cognitive decline can occur in VP at presentation or develop early in the disease course. The dementia in VP is subcortical, manifesting as dysexecutive syndrome with impairment of attention, planning, judgment, goal-directed behaviour, abstract thinking, verbal fluency, in association with behavioural problems, especially apathy, which becomes more common and severe in later stages.^1,41-44^

In the study by Zijlmans et al.^13^ of 17 patients with VP, five had some degree of cognitive dysfunction at the time of their initial presentation with Parkinsonism. In two, dementia was later confirmed by neuropsychological testing, while in two others, a frontal lobe syndrome with slowing of thought processes, loss of abstract thinking, apathy and perseveration were seen. The fifth patient had isolated short-term memory impairment. Six other patients developed cognitive decline after disease onset, progressing to dementia in four.

A Croatian study by Bradvica et al.^44^ compared cognitive dysfunctions between PD and VP and showed greater cognitive impairment in the latter group. An Italian study by Santangelo et al.^45^ compared the neuropsychological profile of patients affected by Parkinsonism and vascular lesions to that observed in patients with PD alone and concluded that VP patients, with or without concomitant dopaminergic denervation disclosed on dopamine transporter imaging, performed worse than the PD group on frontal/executive tasks. In an additional study, Lee et al.^37^ found that the presence and severity of white matter lesions in patients with PD was independently associated with cognitive impairment, regardless of age, gender, duration or severity of PD symptoms, or vascular risk factors. However, none of these studies had clinico-pathological correlations.

The PRIAMO study, a cross-sectional longitudinal observational study designed to evaluate non-motor symptoms in patients with different forms of Parkinsonism in various Italian centers, found VP to be the third most common entity to cause non-motor symptoms, after multiple system atrophy and DLB. The three most commonly reported non-motor symptoms were fatigue, psychiatric symptoms, and attention/memory impairment. Sleep disturbances, urinary and gastrointestinal symptoms and pain, in this order, were the next most commonly reported non-motor symptoms in more than half the patients. However, the presence of specific non-motor features did not help differentiate Parkinsonian syndromes, with autonomic features being the exception, favoring the diagnosis of multiple system atrophy.^1,41^ In this study, attention and memory impairment were found in 73.5% of patients with VP after a mean disease duration of 4.4±3.4 years. Mini-Mental State Examination scores were ≤23.8 points in 29% of patients with VP as compared to 12% of patients with PD.^1,41^

In the Bambuí Study,^43^ eight of the 13 VP patients (61.5%) presented the diagnosis of VD, a feature that was more prevalent than in PD cases.

## NEUROIMAGING

Winikates et al.^12^ showed that involvement of multiple vascular territories, periventricular white matter changes, subcortical ischemic white matter changes, and ischemia of the basal ganglia and brainstem, were all significantly more common in the VP group compared with PD. Zijlmans et al.^23^ compared MRI findings in patients with PD, suspected VP, and hypertensive controls. They found a greater volume of subcortical lesions and greater evidence of lesions of the subcortical gray nuclei in patients with suspected VP compared with both PD and hypertensive controls.

According to the systematic review of Kalra et al.,^43^ five of the 25 studies compared brain CT and/or MRI changes between VP and PD. Patients with VP were significantly more likely to have abnormal imaging, ranging from 90 to 100% compared with patients with PD, ranging from 12 to 43%.^11,12,42,46^ The main abnormalities included multiple territory infarcts, periventricular and subcortical white matter lesions and basal ganglia ischemic lesions. Likewise, significantly more subcortical lesions were present in patients with progressive VP compared with PD patients or hypertensive controls.^23^ Another study indicated that brainstem atrophy was more common in patients with VP and progressive supranuclear palsy (PSP) than in subjects with idiopathic PD.^47^ It is noteworthy that up to 10% of patients with VP have normal MRI.

The value of DAT-SPECT has been explored in several studies with different results. Three studies compared presynaptic striatal dopamine transporter SPECT between VP and PD.^36,48,49^ Diagnostic criteria also differed among studies. Significant reduction in striatal uptake ratios was observed in PD when compared to VP. Contrafatto et al.^50^ used the striatal asymmetry index on DAT-SPECT to differentiate VP from PD. The binding of the ligand in the most affected side proved significantly higher in VP compared to PD. A cut-off value of the index greater than 14.08 was able to differentiate PD from VP with 100% specificity and 50% sensitivity. In summary, a normal or mild and symmetrical reduction in [^123I^] FP-CIT uptake supports the diagnosis of VP if clinical criteria are fulfilled and marked cerebrovascular disease or strategic infarction is present on MRI/CT. Strictly unilateral reduced uptake in the region of a defined vascular lesion on MRI/CT can also be considered VP.^51^

## TREATMENT

VP has generally been believed to respond poorly or not at all to L-dopa treatment.^11,12,22,23,42^ In the only systematic clinico-pathological study to date describing L-dopa response, VP was characterized by a negative response to the drug. These authors only examined patients whose Parkinsonism was caused by small vessel disease and not by macroscopic lacunar infarcts in the basal ganglia. Zijlmans et al.^52^ reported that a substantial number of patients with clinically-suspected VP may respond favorably to dopaminergic therapy, especially those with lesions in or close to the nigrostriatal pathway (macroscopically visible lacunar infarcts or lacunae caused by enlarged perivascular spaces in the putamen, caudate nucleus, and globus pallidus, or microscopic substantia nigra loss). They concluded that a good response to L-dopa in their patients was not predicted by onset type or by any dominant features (tremor, hypokinetic rigidity or shuffling gait).

The VADO study suggested that imaging of striatal DAT facilitates patient management whereas a finding of normal uptake is associated with no benefit from medications in over 90% of subjects.

In clinical practice, the authors' experience is that the majority of patients with clinical diagnosis of VP fail to respond to levodopa. Moreover, due to the high frequency of cognitive and behavioral problems in this population, they do not tolerate anti-Parkinsonian agents as well as patients with PD. Clinicians should bear in mind that there is a risk of worsening of confusion, agitation and even postural instability. Nevertheless, the rare cases of patients with VP due to pre-synaptic lesion often have a good response to levodopa albeit with the possibility of developing complications such as fluctuations and dyskinesias.

**Conclusions.** VP is a form of secondary Parkinsonism usually resulting from ischemic cerebrovascular disease, and is potentially confused with PD, LBD, PSP and other causes of the syndrome. Estimates of the frequency of VP vary greatly worldwide; 3% to 6% of all cases of Parkinsonism are found to have a vascular etiology in most surveys. In two Brazilian community-based studies on the prevalence and classification of Parkinsonism in the elderly, 15.1% and 23.8% of all cases were classified as VP, representing the third most common form, with prevalence rates of 1.1% and 2.3%, respectively. The high number of VP patients in Brazil was probably related to the well-established vascular risk factors such as increased age (both studies were in elderly cohorts), hypertension, stroke, diabetes, smoking, leading to strategic infarcts of the subcortical gray matter nuclei, diffuse white matter ischemic lesions and less commonly, large vessel infarcts. Structural and functional neuroimaging have been included in the diagnostic criteria and surveys, enhancing accuracy. Patients with VP tend to be older than those with PD, and present with early gait difficulties, symmetrical predominant lower-body involvement, postural instability and falls, cognitive impairment and dementia. Cognitive decline can occur in VP at presentation or develop early in the disease course. Dementia in VP is usually subcortical, manifesting as dysexecutive syndrome with impairment of attention, planning, judgment, goal directed behavior, abstract thinking, verbal fluency, in association with behavioral problems, especially apathy. VP has generally been believed to respond poorly or not at all to L-dopa treatment. The treatment approach for VP can be problematic, and neurologists and clinicians should emphasize control of comorbidities and vascular risks, and also involve a multidisciplinary team with a physical therapist, speech pathologist and occupational therapist, toward achieving the best functional performance and quality of life for each patient.

## Figures and Tables

**Figure 1:**
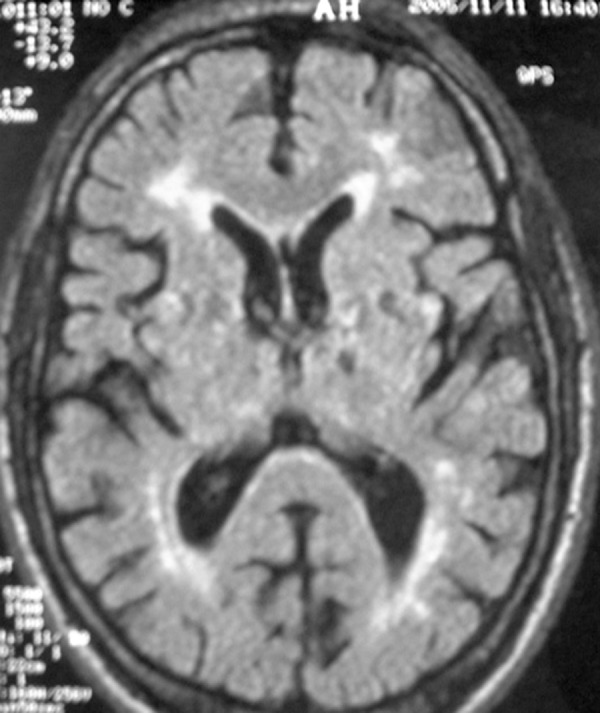
A 68-year-old woman with hypertension and type 2 diabetes presented with delay-onset of progressive Parkinsonism combined with executive dysfunction, emotional incontinence, gait difficulties and pyramidal signs. FLAIR-sequenced brain magnetic resonance imaging showed periventricular white matter abnormalities combined with multilacunar infarcts in the basal ganglia. Patient did not respond to L-dopa therapy.

**Figure 2:**
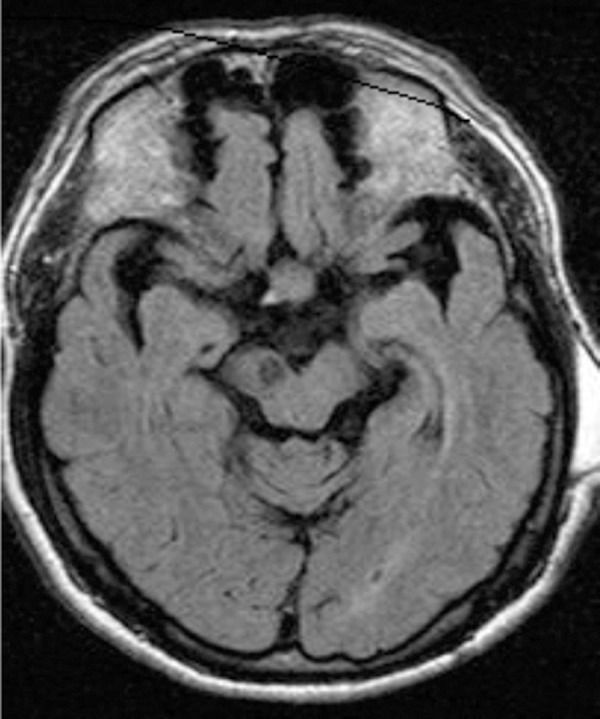
A 57-year-old hypertensive man developed acute-onset left-sided Parkinsonism with postural tremor combined with pyramidal signs. T1- weighted brain magnetic resonance imaging showed a strategic infarct in the right substantia nigra. Patient had a partial response to L-dopa.
